# Reciprocal regulation between *Acinetobacter baumannii* and *Enterobacter cloacae* AdeR homologs: implications for antimicrobial resistance and pathogenesis

**DOI:** 10.1371/journal.pone.0315428

**Published:** 2025-03-10

**Authors:** Marc Gaona, Jordi Corral, Miquel Sánchez−Osuna, Susana Campoy, Jordi Barbé, María Pérez-Varela, Jesús Aranda

**Affiliations:** 1 Departament de Genètica i de Microbiologia, Facultat de Biociènces, Universitat Autònoma de Barcelona (UAB) Campus Bellaterra, Barcelona, Spain; 2 Laboratori de Recerca en Microbiologia i Malalties Infeccioses, Hospital Universitari Parc Taulí, Institut d’Investigació i Innovació Parc Taulí (I3PT−CERCA), UAB Sabadell, Barcelona, Spain; 3 Institut de Biotecnologia i Biomedicina, UAB Campus Bellaterra, Cerdanyola del Vallès, Barcelona, Spain; University of Buea, CAMEROON

## Abstract

*Acinetobacter baumannii* and *Enterobacter cloacae* are phylogenetically distant Gram−negative bacterial pathogens that represent significant challenges in healthcare settings due to their remarkable ability to acquire antimicrobial resistance. This study investigates one of the most important efflux pump systems in *A. baumannii*, AdeABC−AdeRS, and identifies homologous components in *E. cloacae*. By constructing isogenic knockout mutants, we show that the AdeB pump component and the AdeR regulator are significant for antimicrobial resistance and pathogenicity in *A. baumannii*. Through *in silico* predictions, we identify homologs of AdeB and AdeR (ECL_01758 and ECL_01761, respectively) in *E. cloacae*. Notably, we demonstrate that while the inactivation of the *E. cloacae* gene encoding the AdeB protein does not impact on pathogenesis and only alters colistin susceptibility, a knockout mutant of the gene encoding the AdeR regulator significantly affects susceptibility to various antimicrobial classes, motility, and virulence. Additionally, we demonstrate that the AdeR regulators *of A. baumannii* and *E. cloacae* can functionally substitute for each other both *in vitro* and *in vivo* conditions. Electrophoretic mobility shift assays reveal that these regulators are capable of binding to the promoter regions of each other’s species, where similar DNA motifs are present. Furthermore, cross−complementation tests show that the affected phenotypes in each species can be restored interchangeably. Moreover, phylogenomic analysis of previously published *E.cloacae* genomes and reconstructrion of ancestral states through the phylogenetic trees of the *adeB* and *adeR* genes suggest that these homologs are more likely derived from a common ancestor rather than through recent horizontal gene transfer. The findings of this work highlight that conserved regulatory functions concerning efflux pump expression can be maintained across species despite evolutionary divergence and open new perspectives for the control of bacterial infections.

## Introduction

In recent years, the global healthcare scenario has faced substantial challenges due to the increasing emergence of antimicrobial–resistant bacterial pathogens. In particular, Gram−negative bacteria have gained notoriety for their capacity to induce severe infections within healthcare settings, leading to prolonged hospitalizations, elevated mortality rates, and increased economic burdens [1]. Among these microorganisms, *Acinetobacter baumannii*, a ubiquitous environmental bacterial species, has emerged as an important opportunistic pathogen responsible for a diverse range of healthcare–associated infections, including pneumonia, bacteremia, urinary tract infections, and wound infections [2].

Concurrently, various species within the *Enterobacteriaceae* family, such as *Enterobacter* spp., present a significant threat as nosocomial pathogens with the potential to cause illnesses ranging from urinary tract to bloodstream infections [3]. These bacterial species have demonstrated remarkable adaptability to diverse environments and have developed intricate resistance mechanisms against commonly used antimicrobial agents, thereby presenting a challenge to the efficacy of conventional treatment strategies.

Among the antimicrobial resistance mechanisms, efflux pumps play an important role in bacterial pathogens [4–7]. The primary role of efflux pumps is to remove any potentially dangerous molecules from the inside of the bacterial cell. Nevertheless, in recent years, it has been found that they also play a significant role in several processes related to bacterial pathogenesis. For instance, the secretion of surfactants involved in motility [4] or the extrusion of molecules related to quorum sensing and biofilm formation [5]. In addition, efflux pumps can act as virulence factors by themselves [6] or can secrete them [7].

RND (Resistance Nodulation Division) is one of the most significant superfamilies of efflux pumps related to antimicrobial resistance, allowing the emergence of multidrug–resistant (MDR) bacteria, extensively drug–resistant (XDR) bacteria, and even pandrug–resistant (PDR) bacteria that show resistance to all antibacterial classes [8]. The RND efflux pumps are found primarily in Gram–negative bacteria, which exhibit a more complex cell envelope structure compared to Gram–positive bacteria. This complexity provides an additional barrier, the cell outer membrane, that antimicrobials must traverse, making efflux pumps in Gram–negative bacteria particularly effective as resistance mechanisms [9].

In *A. baumannii*, the AdeABC RND (regulated by AdeRS) is one of the most important efflux pumps involved in antimicrobial resistance, including β–lactams, fluoroquinolones, tetracycline, tigecycline, macrolide, aminoglycosides [10]. It is important to note that the underlying mechanism of colistin resistance may result from specific alterations in AdeRS, which lead to the overexpression of AdeAB [11,12]. The *adeABC* operon codifies the three RND components: (i) the inner membrane transporter AdeB, (ii) the membrane fusion protein AdeA, and (iii) the outer membrane protein AdeC [13]. AdeRS is a TCS (Two–Component System) codified upstream and in opposite direction of the *adeABC* operon, where the kinase sensor (AdeS) receives specific environmental signals and phosphorylates the response regulator AdeR, which specifically binds to the intercistronic spacer region between *adeR* and *adeA* genes, thereby increasing the *adeABC* expression [14].

To our knowledge, no homologs of the *A. baumannii* AdeABC–AdeRS proteins have been identified in *Enterobacteriaceae* to date. The best–characterized RND efflux pump within species belonging to this family of Gram–negative bacteria is AcrAB–TolC [15]. In this efflux pump, AcrA, AcrB, and TolC are the periplasmic adaptor, the inner membrane transporter, and the outer membrane proteins, respectively. AcrAB–TolC is considered the most clinically important efflux pump not only in enterobacteria but also in other Gram−negative species [16].

In *Enterobacter cloacae*, a representative species of *Enterobacteriaceae*, the AcrAB–TolC system is the RND with a major role not only in resistance to antimicrobials but also in pathogenicity, although active efflux remains poorly characterized [17]. Its expression is under the direct control of various transcriptional regulators, with the AcrR repressor (belonging to the TetR family of transcriptional regulators) being the main one [18].

In this study, we identify homologs of the main components of the AdeABC–AdeRS system from *Acinetobacter baumannii* in *Enterobacter cloacae*. We analyzed their regulation and implications for antimicrobial resistance and pathogenesis in both species. Furthermore, we investigated the functional interchangeability between the regulators of these efflux systems in both pathogens. Additionally, we conducted *in silico* predictions of *A. baumannii* AdeB and AdeR homologs and performed ancestral reconstruction analysis of the *adeB* and *adeR* genes in *E. cloacae* to explore the hypothesis that these genes originated from a common ancestor shared by *Acinetobacter* and *Enterobacter* species.

## Results

### The inner membrane RND transporter AdeB and the response regulator AdeR are involved in the antimicrobial resistance, surface–associated motility and virulence of *A. baumannii* ATCC 17978 strain

Inactivation of the *A. baumannii* ATCC 17978 (GenBank: CP018664.1) genes encoding the inner membrane transporter AdeB (*AUO97_16535*, protein_id = “APP32353”) or the response regulator AdeR (*AUO97_16545*, protein_id=” APP32355”), respectively referred to as AdeB_Ab_ and AdeR_Ab_, led to a 2 − to 4 − fold increase in susceptibility to a range of antibacterial compounds (Table 1). These findings confirm that the coordinated function of AdeB_Ab_ and AdeR_Ab_ plays an important role in the development of antimicrobial resistance.

**Table 1:** Minimum inhibitory concentrations (MICs, mg/L) of the indicated antimicrobials for wild−type *A. baumannii* ATCC 17978 (WT) and derivative mutants lacking either the *adeR*__*Ab*__ or *adeB*__*Ab*__ genes.

Antimicrobial	WT	*ade* *B*__*A*b__	*ade* *R*__*A*b__
Ceftazidime	4	4	2
Rifampin	4	2	2
Tetracycline	1	0.5	1
Amikacin	2	0.5	1
Gentamicin	1	0.25	0.5
Erythromycin	2	1	1
Chlorhexidine	4	1	4
Ciprofloxacin	0.25	0. 25	0.125
Levofloxacin	0.032	0.032	0.016
Minocycline	0.25	0.125	0.125
Trimethoprim	16	4	4
Colistin	1	1	1
Imipenem	0.125	0.0625	0.0625
Tigecycline	0.25	0.0625	0.0625

**Fig 1:**
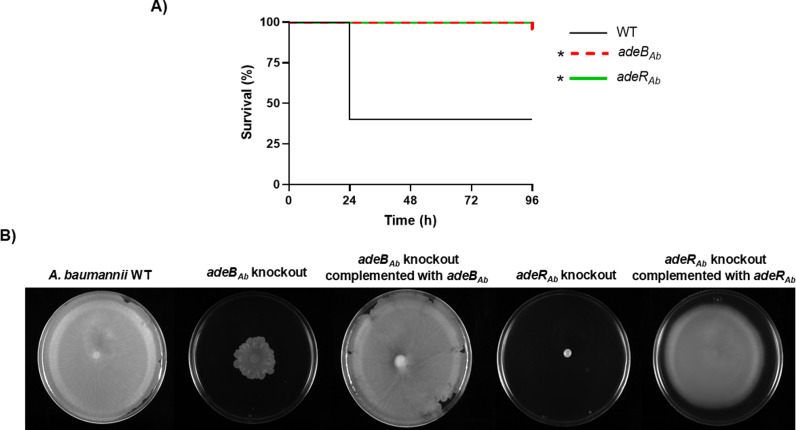
*A. baumannii* virulence and motility assays. **(A)** *G. mellonella* killing assay of the specified strains. Larvae (n =  10 per group) were inoculated with either ~ 10^6^ CFU of the indicated strain or PBS as a negative control (data not shown). * *P* < 0.005 compared to the *A. baumannii* parental ATCC 17978 strain (WT). All *G. mellonella* killing experiments were performed at least twice obtaining reproducible results. A representative graph of larval survival is shown. **(B)** Motility assays of wild−type *A. baumannii* strain ATCC 17978 (WT) and the indicated derivatives. All motility assays were performed at least three times obtaining reproducible results. A representative image is shown.

We also observed that disrupting either the *adeB*_*Ab*_ or *adeR*_*Ab*_ genes reduced lethality in the wax worm model (Fig 1A). Interestingly, both knockouts exhibited impaired surface−associated motility, with the effect being more pronounced in the *adeR*_*Ab*_ knockout (Fig 1B). As expected, reintroducing the wild−type genes completely restored motility (Fig 1B).

### Homologs of AdeB and AdeR in *Enterobacter* cloacae

A BLASTP analysis was conducted to identify putative homologs of the AdeB_Ab_ and AdeR_Ab_ proteins from *A. baumannii* ATCC 17978 in the *E. cloacae* subsp. *cloacae* ATCC 13047 genome (GenBank: CP001918.1). This analysis identified ECL_01758 (GenBank: ADF61316; referred to as AdeB_Ec_) as the reciprocal best hit for AdeB_Ab_, with 58.1% sequence identity and 97.0% coverage. Additionally, ECL_01761 (GenBank: ADF61319; referred to as AdeR_Ec_) was identified as the AdeR_Ab_ homolog in *E. cloacae*, showing 47.8% sequence identity and 91.0% coverage.

**Fig 2:**
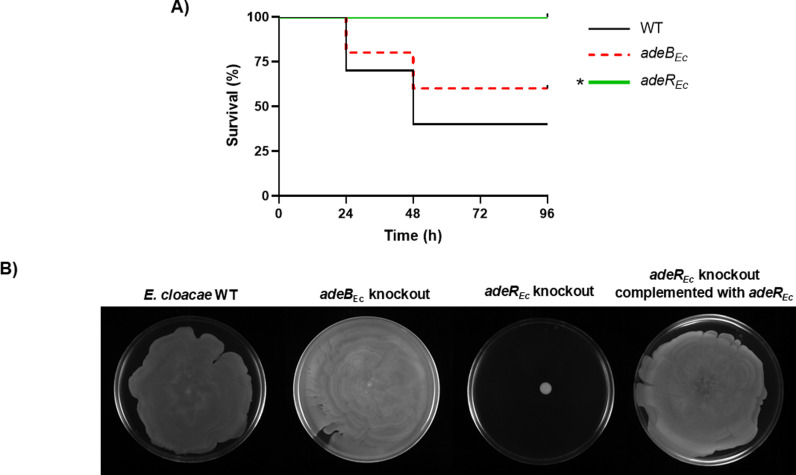
*E.* *cloacae* virulence and motility assays. **(A)** *G. mellonella* killing assay of the specified strains. Larvae (n =  10 per group) were inoculated with either ~ 10^8^ CFU of the indicated strain or PBS as a negative control (data not shown). * *P* < 0.05 compared to the *E. cloacae* parental ATCC 13047 strain (WT). All *G. mellonella* killing experiments were performed at least twice obtaining reproducible results. A representative graph of larval survival is shown. **(B)** Motility assays of wild−type *E. cloacae* strain ATCC 13047 (WT) and the indicated derivatives. All motility assays were performed at least three times obtaining reproducible results. A representative image is shown.

Phenotypic analysis of the *adeB*_*Ec*_ knockout mutant generated in this study revealed no significant changes in virulence or motility (Fig 2), nor in antimicrobial susceptibility, except for colistin (Table 2). Notably, the *adeR*_*Ec*_ knockout mutant constructed in this work exhibited altered susceptibility to various antimicrobial classes (Table 2), a significant reduction in virulence in the wax worm model (Fig 2A), along with a complete loss of motility (Fig 2B), which was fully restored by complementation (Fig 2B). These findings suggest that, similar to its role in *A. baumannii*, the AdeR_Ec_ regulator is involved in controlling other genes associated with these phenotypes.

**Table 2:** Minimum inhibitory concentrations (MICs, mg/L) of the indicated antimicrobials for wild−type *E. cloacae* ATCC 13047 (WT) and derivative mutants lacking either the *adeR*__*Ec*__ or *adeB*__*Ec*__ genes.

Antimicrobial	WT	*ade* *B*__*E*c__	*ade* *R*__*E*c__
Ceftazidime	4	4	64
Cefotaxime	8	8	32
Rifampicin	32	32	16
Tetracycline	2	2	1
Chloramphenicol	2	2	1
Minocycline	4	4	2
Levofloxacin	0.016	0.016	0.008
Ciprofloxacin	0.016	0.016	0.008
Colistin	512	16	16
Imipenem	0.5	0.5	0.5
Tigecycline	0.5	0.5	0.5

### *A. baumannii* and *E. cloacae* AdeR homologs are functionally interchangeable

**Fig 3:**
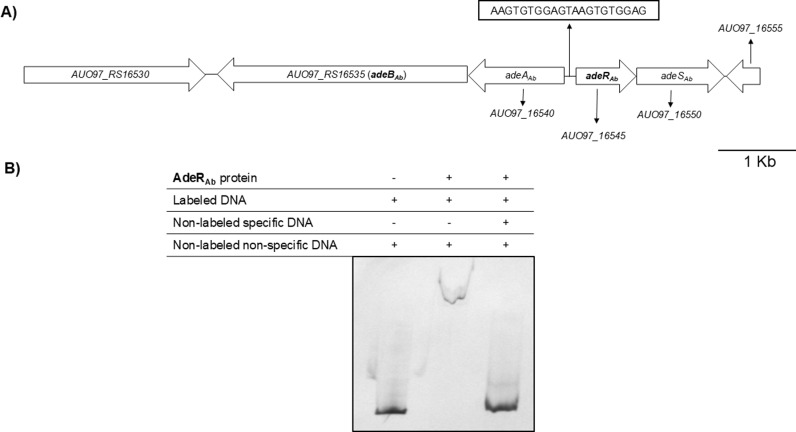
Genetic surroundings of *adeAB*__*Ab*__ and *adeR*__*Ab*__ genes demonstrating AdeR_Ab_ binding to their intergenic region. **(A)** Intergenic region showing the DNA motif found in the 136 bp between the *adeAB*_*Ab*_ operon and the *adeR*_*Ab*_ gene in *A. baumannii* strain ATCC 17978. The surrounding genes and the conserved direct repeat motif are also shown. **(B)** EMSA of a DIG−labeled DNA fragment containing the 136 − bp intercistronic region. The experiment was carried out in the presence (+) or absence (−) of 2 μM of AdeR protein, with at least a 10 − fold molar excess of non−labeled and non−specific DNA in all lanes, and non−labeled and specific DNA (third lane). All EMSAs were performed at least three times obtaining reproducible results. A representative image is shown.

As part of a two−component regulatory system with the sensor AdeS, AdeR regulates the expression of the AdeABC pump in *A. baumannii* by binding to a conserved direct repeat motif within a 133 − bp (base pairs) intergenic region, as observed in the *A. baumannii* AB260 and AB293 clinical isolates [19]. Sequence analysis of the corresponding chromosomal region in the *A. baumannii* ATCC 17978 strain revealed a similar 136 − bp intercistronic region to which the AdeR_Ab_ protein specifically binds (Fig 3).

**Fig 4:**
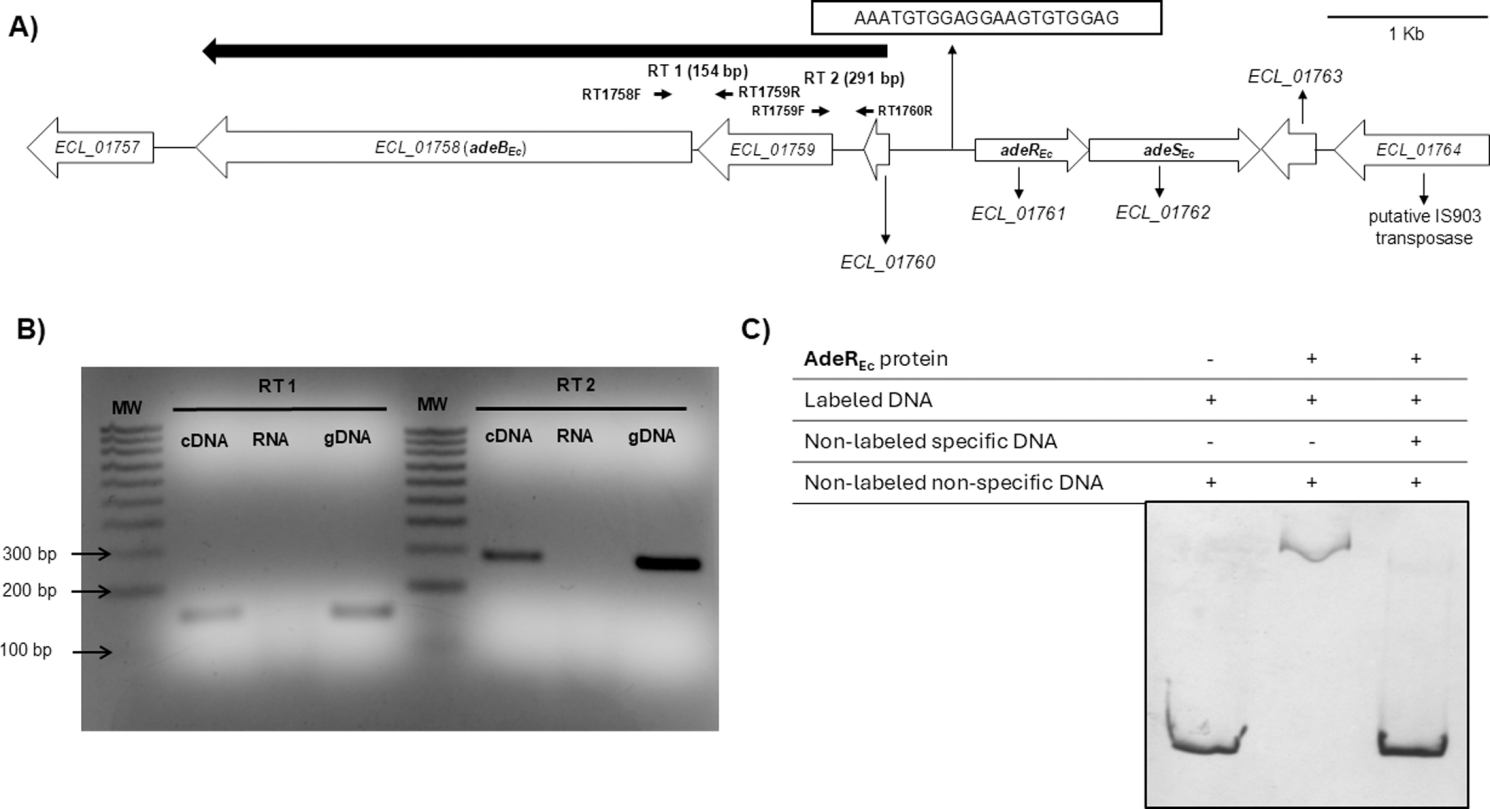
Genetic surroundings of *adeB*__*Ec*__ and *adeR*__*Ec*__ genes demonstrating AdeR_Ec_ binding to their intergenic region. **(A)** Genetic organization of the chromosomal region in *E. cloacae* ATCC 13047 containing the genes encoding the AdeR_Ec_ and AdeB_Ec_ proteins, as determined by RT−PCR analysis of RNA. The large, filled arrow represents the transcriptional unit analyzed. The primer sets used for the RT−PCR analyses are indicated by small arrows. The conserved DNA motif found is also shown. **(B)** RT−PCRs were performed in the presence of DNA (lane A), total RNA (lane B), and gDNA (lane C). The NZYDNA Ladder V (NZYtech) was used as a molecular size marker (lane MW). **(C)** EMSA of a DIG−labeled DNA fragment containing the 539 − bp intercistronic region with the DNA motif. The experiment was carried out in the presence (+) or absence (−) of 2 μM of the *E. cloacae* AdeR_Ec_ protein, with at least a 10 − fold molar excess of non−labeled and non−specific DNA in all lanes, and non−labeled and specific DNA (third lane). All EMSAs were performed at least three times obtaining reproducible results. A representative image is shown.

In *A. baumannii*, the *adeRS* genes are encoded upstream and in the opposite direction of the genes coding for the RND AdeA and AdeB proteins (Fig 3). In this species, two components of the RND pump are organized together in a single operon. In contrast, in *E. cloacae*, only the permease component of the RND system is present, along with two other genes encoding the ECL_01759 and ECL_01760 transporters, which do not belong to the RND family (Fig 4A) and are involved in resistance pathways for NOSO**–**502 and colistin [20].

Our RT−PCR analysis confirmed that *adeB*_*Ec*_ is transcribed with *ECL_01759* and *ECL_01760* as part of the same transcriptional unit (Fig 4B). Notably, recent RT − qPCR data have shown that the product of the *ECL_01761* gene (*adeR*_*Ec*_) functions as an activator controlling their expression [20]. For this reason, the sequence of the 539 − bp intergenic region between the *adeR*_*EC*_ gene and the preceding gene (*ECL_01760*) was analyzed. This analysis revealed a DNA motif like that of *A. baumannii* located 159 bp upstream of the ORF (Open–Reading Frame) encoding the *E. cloacae* AdeR_EC_ protein (Fig 4A).

**Fig 5:**
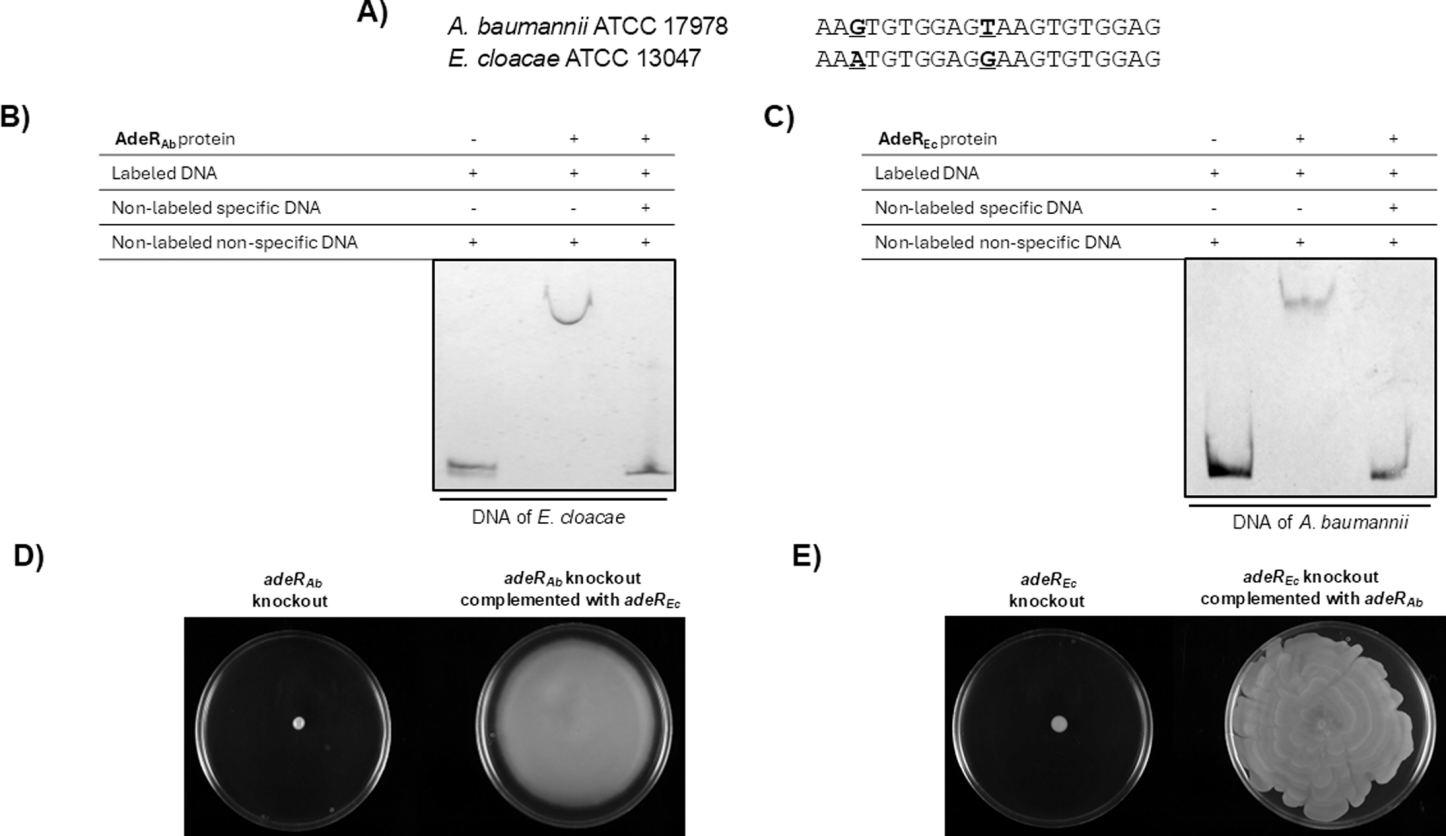
*In vitro* and *in vivo* interchangeability of *Acinetobacter* and *Enterobacter* regulators. **(A)** Alignment of direct repeat motifs found in the intercistronic regions of *A. baumannii* ATCC 17978 and *E. cloacae* ATCC 13047. Nucleotide changes are highlighted in bold and underlined. **(B &** **C**) EMSAs of a DIG−labeled DNA fragments containing the 539 − bp *E. cloacae* (B) and the 136 − bp *A. baumannii* (C) intercistronic regions. The experiments were carried out in the presence (+) or absence (–) of 2 μM of the indicated AdeR protein, with at least a 10 − fold molar excess of non−labeled and non−specific DNA in all lanes, and non−labeled and specific DNA (third lane). All EMSAs were performed at least three times obtaining reproducible results. Representative images are shown. **(D & E)** Motility assays of the indicated *A. baumannii* (D) and *E. cloacae* (E) strains. All motility assays were performed at least three times obtaining reproducible results. Representative images are shown.

As expected, electrophoretic mobility shift assays (EMSAs) confirmed that the *E. cloacae* AdeR_Ec_ protein specifically binds the promoter region containing the identified DNA motif (Fig 4C). Furthermore, interspecies EMSAs revealed reciprocal binding interactions: the AdeR_Ec_ transcriptional regulator from *E. cloacae* binds to the promoter region of the operon encoding the *A. baumannii* efflux pump AdeABC, and conversely, the *A. baumannii* AdeR_Ab_ regulator binds to the promoter region of the genes encoding the *E. cloacae* transporters (Fig 5A − C).

This reciprocal interaction was further supported by *in vivo* phenotypic complementation experiments, where the introduction of the gene encoding the *E. cloacae* transcriptional regulator AdeR_Ec_ restored affected phenotypes in the *A. baumannii adeR*_*Ab*_ knockout mutant (Fig 5D). Conversely, the introduction of the gene encoding the *A. baumannii* AdeR_Ab_ transcriptional regulator restored altered phenotypes in the corresponding *E. cloacae* knockout mutant (Fig 5E).

### Evolutionary history of the *Acinetobacter* and *Enterobacter* AdeB and AdeR homologs

The *E. cloacae adeR*_*Ec*_ gene is located near a putative transposase gene, *ECL_01764* (Fig 4A), prompting questions about its genomic context and evolutionary history. To investigate this, we conducted a comprehensive phylogenetic analysis using genomic data from the NCBI database. Our analysis focused on the presence of genes encoding homologs of AdeR and AdeB in publicly available *Gammaproteobacteria* complete assemblies, encompassing all genomes of *Acinetobacter* and *Enterobacter* species (Tables S1–S3).

The results obtained revealed a high prevalence of these genes, ranging from 81.4% to 95.3%, in *A. baumannii* and *Acinetobacter pittii* genomes (Table 3; detailed information is available in Table S1). In the *Enterobacter* genus, specifically *E. cloacae*, there was a moderate presence, with a prevalence of 52.9% for AdeB homologs and 54.3% for AdeR homologs (Table 3; detailed information is available in Table S2).

**Table 3:** Prevalence of *adeR* and *adeB* genes in representative *Acinetobacter* and *Enterobacter* species (n =  number of analyzed genomes).

*Acinetobacter* spp.	n	*adeR* (%)	*adeB* (%)	*Enterobacter* spp.	n	*adeR* (%)	*adeB* (%)
*A. baumannii*	575	88	91.8	*E. hormaechei*	258	0	0
*A. pittii*	43	95.3	81.4	*E. cloacae*	70	54.3	52.9

*adeB* and *adeR* nucleotide identities between *A. baumannii* ATCC 17978 and *E. cloacae* ATCC 13047 are 57.61% and 51.79%, respectively.

**Fig 6:**
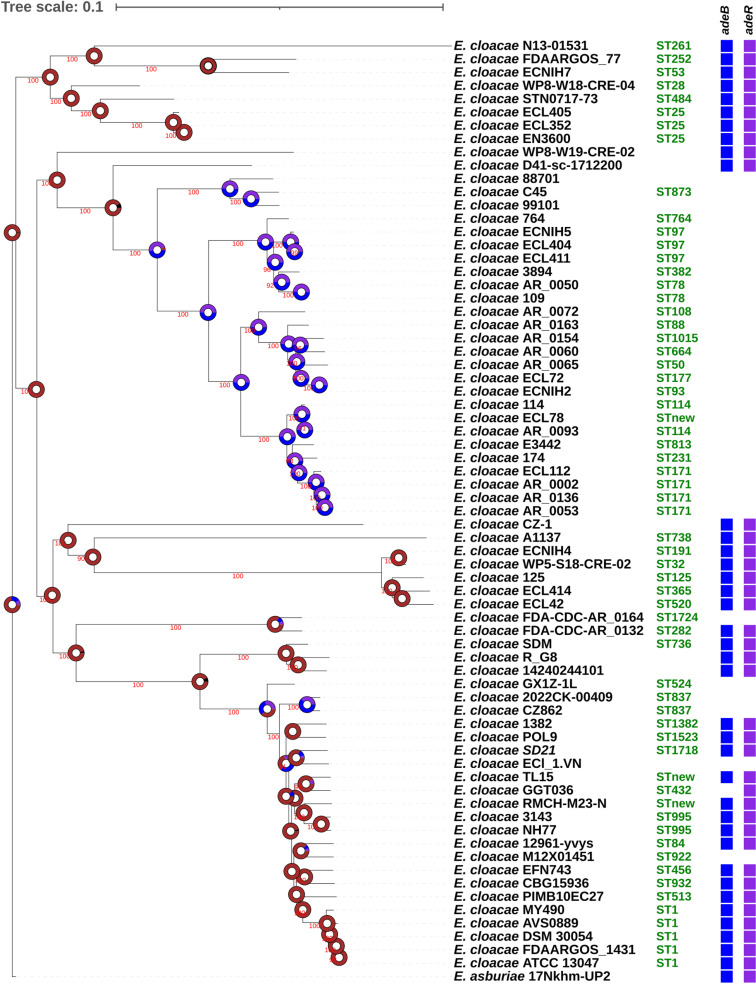
ML core genome phylogenetic analysis of all analyzed *E. cloacae* sequences. Branch support values are provided as the percentage of bootstrap replicates in which the branching was observed. Support values are only shown for branches with at least 75% support. Next to each tip label, colored boxes indicate presence of *adeB* and/or *adeR* genes. Reconstructed presence of *adeB* (blue), *adeR* (purple) or both (maroon) are displayed on nodes as colored charts. *Enterobacter asburiae* 17Nkhm–UP2 genome was used as the root.

A phylogenetic analysis of all analyzed *E. cloacae* sequences indicated that the presence of AdeR and AdeB in this species is likely ancestral and that most of the genomes lacking AdeR and AdeB homologs forms a well−supported cluster, suggesting a single loss event in these genomes. However, as all strains in this cluster lack both genes, the ancestral state reconstruction could not establish whether one gene was lost before (Fig 6). No homologs for AdeB and AdeR genes were predicted in *Enterobacter hormaechei* genomes (n = 258). Finally, AdeB and AdeR homologs were found in other *Gammaproteobacteria* outside the *Enterobacter* and *Acinetobacter* genera (Table S3), a fact that would support an ancestral heritage.

**Fig 7:**
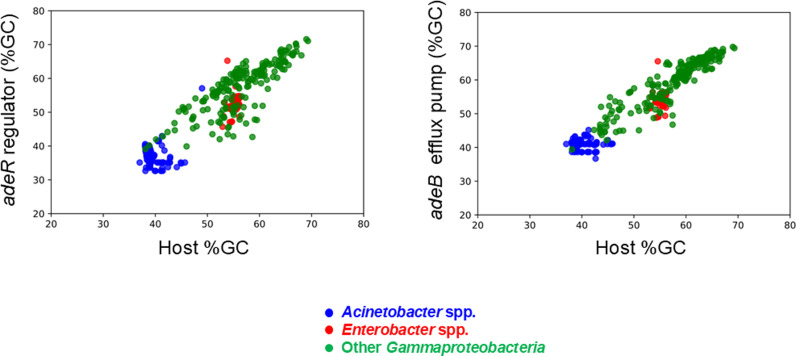
Percentage of GC content of the *adeR* and *adeB* genes. Scatter plots showing the correlation between the %GC content of the *adeR* (left) and *adeB* (right) genes and that of their host chromosomes.

The assessment of %GC content is a useful approach for elucidating evolutionary dynamics and detecting potential recent lateral gene transfer events. Comparing the %GC content of specific genes with that of their host genome could highlight mobilization events, as previously described [21]. The results presented in Fig 7 demonstrate that the %GC content of *adeB* (Pearson Correlation Coefficient, PCC =  0.9) and *adeR* (PCC =  0.8) homologs is well−aligned with the %GC content of their host genomes. Specifically, the coding sequences for *E. cloacae* AdeR_Ec_ and AdeB_Ec_ proteins exhibited higher GC content (52.8% ±  2.1 SD for *adeR*_*Ec*_ and 54.6% ±  1.8 SD for *adeB*_*Ec*_) compared to *adeR*_*Ab*_ and *adeB*_*Ab*_ of *A. baumannii* (36.5% ±  1.4 SD for *adeR*_*Ab*_ and 40.8% ±  0.7 SD for *adeB*_*Ab*_). These findings strongly suggest that the observed differences in %GC content is likely due to divergent evolution from a shared common ancestry rather than recent horizontal gene transfer events.

## Discussion

In recent decades, efflux systems have become a major challenge in medicine, as pathogenic bacteria have increasingly developed resistance to multiple drugs, likely driven by the widespread use of antibiotics and antimicrobial agents. Many efflux pumps, particularly those in the RND superfamily, are crucial contributors to clinically significant antibiotic resistance, highlighting the importance of understanding their broader biological roles.

In *Escherichia coli*, approximately 80% of efflux pumps are conserved across different phylogroups, suggesting that efflux−encoding genes are a core part of the genome [22]. The primary role of these pumps is to safeguard cells from toxic substances, including those produced by the bacteria themselves. Many known antibiotics were initially isolated from actinomycetes, especially the genus *Streptomyces*. In these antibiotic−producing organisms, efflux systems likely protect the cells from their own bioactive secondary metabolites [23]. However, the conservation of efflux pumps among diverse bacterial species indicates that these genes have functions beyond antimicrobial resistance, potentially including roles in cellular detoxification, survival in hostile environments, cell−to−cell communication, biofilm formation, and virulence [22].

In *A. baumannii*, the AdeABC RND efflux pump, regulated by the AdeRS system, is one of the most significant mechanisms contributing to antimicrobial resistance [10]. Compared to the wild–type (WT) parental strain, isogenic knockout mutants of both *adeB*_*Ab*_ and *adeR*_*Ab*_ genes had 2– to 4–fold higher susceptibilities to aminoglycosides, beta–lactams, tetracyclines, rifampin, erythromycin, quinolones, and trimethoprim, among other antimicrobial agents, showing similar results that those previously reported [13,14,24,25]. Interestingly, efflux pumps like AdeABC have been associated not only with antibiotic resistance but also with other cellular processes, such as motility, as we have demonstrated for both *adeB*_*Ab*_ and *adeR*_*Ab*_ knockout mutants. While both mutants exhibited similar results regarding reduced lethality in the wax worm model, the mutant lacking *adeR*_*Ab*_ demonstrated a more significant reduction in surface–associated motility compared to the *adeB*_*Ab*_ knockout. This observation is noteworthy considering AdeB’s role as an efflux pump and AdeR’s function as a transcriptional regulator. It is important to highlight the work of López *et al*., who specifically investigated the *adeB*_*Ab*_ mutant in the ATCC 17978 strain and similarly reported a loss of motility [25].

The motility of *A. baumannii* depends on the secretion of compounds via RND efflux pumps making it logical to extend this association to the regulators of these efflux pumps [26–28]. This connection is particularly relevant because the AdeRS system, which regulates the AdeABC efflux pump, has also been shown to impact motility and biofilm formation in *A. baumannii* ATCC 17978 strain [29]. Nevertheless, these authors, in contrast to our findings, do not report a significant loss of virulence. This discrepancy may be due to differences in the methodologies used to assess virulence.

Recent studies also reported a strain−dependent variation in virulence, indicating that the expression and functional impact of the AdeABC efflux pump system can differ significantly among *A. baumannii* strains [24]. This variability might be due to differences in selective pressures and ecological niches that these bacteria occupy, which in turn could influence the conservation and functionality of these genes. Likewise, it has been observed a comparable loss of virulence in the *adeAB* mutant from *the A. baumannii* S1 strain, but reported only a slight, nonsignificant change in virulence in an *adeRS* mutant of the *A. baumannii* AYE strain [24]. It is important to note that variations in the expression of the AdeABC efflux pump, regulated by AdeRS, may exist depending on the strain analyzed [30]. Nonetheless, according to the phenotype of the mutant constructed in our study, previous RNAseq analysis has shown that the loss of AdeRS not only decreases the expression of efflux pumps, including AdeABC, but also of genes involved in motility and virulence in the *A. baumannii* AYE strain [24].

Through bioinformatics studies, we have identified homologs of AdeB and AdeR proteins in *E. cloacae*. Phenotypic analysis of the mutant lacking the gene encoding AdeB_Ec_ revealed no significant alterations in motility or virulence, nor in antimicrobial susceptibility, except for colistin, a consistency with the findings previously reported [17,20]. However, we have demonstrated for the first time that the regulator AdeR_EC_ plays a role in resistance to different classes of antimicrobials, as well as in motility and virulence. Accordingly, AcrR, the primary transcriptional repressor of the AcrAB–TolC efflux pump in enterobacteria, has been shown to suppress bacterial motility in *E. coli* by affecting the expression of flagellum biosynthesis and motility genes [31].

In *A. baumannii*, the AdeRS genes regulate the AdeABC efflux pump, which is organized in a single operon, suggesting coordinated regulation and resistance function. Conversely, in *E. cloacae*, only the permease component of the RND system is present, alongside ECL_01759 and ECL_01760, which are not part of the RND family but are involved in resistance to NOSO − 502 and colistin [20]. RT−PCR analysis shows these genes are transcribed in the same mRNA, and RT − qPCR data reveal that the *adeR*_*Ec*_ gene product activates their expression [20].

We have found a conserved DNA motif upstream of the gene encoding the AdeR_Ec_ protein of *E. cloacae*, like one in *A. baumannii*, suggesting a shared regulatory mechanism. The conservation of the AdeRS regulatory system in *E. cloacae*, as demonstrated by the functional interchangeability of AdeR homologs from both species, underscores the robustness and adaptability of these regulatory mechanisms. Despite the significant evolutionary distance between *A. baumannii* and *E. cloacae*, the ability of AdeR_Ab_ and AdeR_Ec_ to bind similar DNA motifs and complement the corresponding phenotypes in knockout mutants indicates a remarkable functional conservation.

Transcriptional regulators are important for bacterial adaptability and survival, as they control gene expression by specifically interacting with DNA sequences. Our study highlights the remarkable functional interchangeability of the AdeR homologs from *A. baumannii* and *E. cloacae*, despite the significant evolutionary divergence between these species. A classic example of such conservation is the LexA repressor. In the SOS response to DNA damage, LexA from various bacterial species can recognize and bind to the SOS box in *E. coli*, illustrating how essential regulatory functions can be preserved across different bacterial lineages [32]. This suggests that, although species−specific adaptations may result in diverse regulatory mechanisms, the fundamental principles of DNA recognition and gene activation can be remarkably conserved across different bacterial species.

The presence of AdeB and AdeR homologs in other *Gammaproteobacteria* indicates a common ancestral origin, suggesting that these genes may have been conserved due to their essential role in bacterial survival and adaptability. The lack of these homologs in species like *E. hormaechei* may reflect unique evolutionary pathways or functional replacements that do not share DNA homology. These data indicate that while several *Enterobacter* species possess homologs of the *A. baumannii* AdeR system, they show less conservation of this system compared to *Acinetobacter*. This suggests that despite both genera may share a common ancestor with these genes, *Enterobacter* species have experienced distinct evolutionary pressures leading to the loss of some of these genes. Differences in selective pressures and ecological niches between *Acinetobacter* and *Enterobacter* could explain this divergence.

Our study demonstrates that the *adeR* homologs in *A. baumannii* and *E. cloacae*, despite the observed sequence divergence (nucleotide identity of 51.79%), share similar regulatory mechanisms. Both species possess AdeR proteins that bind to conserved DNA motifs and can function interchangeably across species, indicating a common molecular basis. The widespread presence of efflux pump genes in bacterial genomes facilitates their transfer through mobilizable plasmids, even among bacteria from different ecological niches [33]. This potential for horizontal gene transfer underscores the risk of widespread dissemination of antimicrobial resistance traits [34]. Besides drug resistance, efflux pumps are also involved in various essential physiological functions such as bacterial adaptation, toxin efflux, biofilm formation, and quorum sensing, with RND superfamily pumps being particularly significant in Gram−negative bacteria [35].

The findings of this study provide significant insights into the role of the AdeABC−AdeRS efflux pump system in *A. baumannii* and its homologs in *E. cloacae*, highlighting the interplay between antimicrobial resistance, motility, and virulence. Additionally, the study shows that the regulatory components are functionally conserved across these phylogenetically distant species, despite their ecological and evolutionary divergence. Understanding the differences in efflux pump systems and their regulation among various bacterial species provides valuable insights into the evolution of antimicrobial resistance. This knowledge can inform the development of new strategies to combat multidrug−resistant bacteria by targeting conserved regulatory functions across different species. The findings of this study emphasize the potential for conserved regulatory mechanisms to be targeted for therapeutic intervention, providing a promising avenue for the development of broad−spectrum antimicrobial agents.

## Materials and methods

### Bacterial strains, plasmids and growth conditions

The bacterial strains and plasmids used in this work are listed in Table 4. *A. baumannii*, *E. coli* and *E. cloacae* strains were grown at 37°C in Luria−Bertani (LB) medium with shaking at 180 rpm. When needed, antimicrobials were added at suitable concentrations: kanamycin 50 mg/L, apramycin 30 mg/L, gentamicin 20 mg/L for *E. coli* and *E. cloacae* and 40 mg/L for *A. baumannii* strains.

**Table 4:** Bacterial strains and plasmids used in this work.

Strain or plasmid	Relevant characteristics	Source or reference
*E. coli* strains		
DH5α	*E. coli supE4 ΔlacU169 (*ɸ*80 ΔlacZ ΔM15) hsdR17, recA1, endA1, gyrA96, thi − 1, relA1*	Clontech
BL21 − CodonPlus(DE3) − RIL	F^–^ *ompT hsdSB(rB − rB) dcm galλ(DE3)*	Clontech
*A. baumannii* strains		
ATCC 17978	Wild−type strain	ATCC
*AUO97_16545* (*adeR*_*Ab*_)	ATCC 17978 derivative strain with *AUO97_16545*::pCR−BluntII−TOPO disruption, Km^r^, Zeo^r^	This study
*AUO97_16535* (*adeB*_*Ab*_)	ATCC 17978 derivative strain with *AUO97_16535*::pCR−BluntII−TOPO disruption, Km^r^, Zeo^r^	This study
*AUO97_16545* (*adeR*_*Ab*_) *glmS:: AUO97_16545* (*adeR*_*Ab*_)	*AUO97_16545* (*adeR*_*Ab*_) strain complemented strain with *AUO97_16545* (*adeR*_*Ab*_) *A. baumannii* wild−type copy inserted into *glmS* gene, Km^r^, Zeo^r^, Apra^r^	This study
*AUO97_16545* (*adeR*_*Ab*_) *glmS::ECL_01761*(*adeR*_*Ec*_)	*AUO97_16545* (*adeR*_*Ab*_) strain complemented strain with *E. cloacae ECL_01761* (*adeR*__***E****c*__) wild−type copy inserted into *glmS* gene, Km^r^, Zeo^r^, Apra^r^	This study
*AUO97_16535 (adeB*_*Ab*_) pBAV1Gm − T5 − gfp Ω *AUO97_16535 (adeB*_*Ab*_)	*AUO97_16535* (*adeB*_*Ab*_) strain carrying pBAV1Gm − T5 − gfp plasmid with *AUO97_16535* (*adeB*_*Ab*_) cloned, Km^r^, Zeo^r^, Gm^r^	This study
*E. cloacae* strains		
ATCC 13047	Wild−type strain	ATCC
*ECL_01761* (*adeR*_*Ec*_)	ATCC 13047 Δ*ECL_01761* (*adeR*__***E****c*__)	This study
*ECL_01758* (*adeB*_*Ec*_)	ATCC 13047 Δ*ECL_01758* (*adeB*_*Ec*_)	This study
*ECL_01761* (*adeR*_*Ec*_) *glmS:: ECL_01761* (*adeR*_*Ec*_)	*ECL_01761* (*adeR*__***E***____*c*__) knockout complemented with *E. cloacae ECL_01761* (*adeR*__***E****c*__) wild−type copy inserted into *glmS* gene, Apra^r^	This study
*ECL_01761* (*adeR*_*Ec*_) *glmS:: AUO97_16545* (*adeR*_*Ab*_)	*ECL_01761* (*adeR*__***E****c*__) knockout complemented strain with *A. baumannii AUO97_16545* (*adeR*_*Ab*_) wild−type copy inserted into *glmS* gene, Apra^r^	This study
*ECL_01758* (*adeB*_*Ec*_) pUA1108Ω *ECL_01758* (*adeB*_*Ec*_)	*ECL_01758* (*adeB*_*Ec*_) knockout carrying pUA1108 plasmid with *ECL_01758* (*adeB*_*Ec*_) cloned, Km^r^	This study
Plasmids		
pBAV1Gm − T5 − gfp	pBAV1K − T5 − gfp derivative complementation vector carrying gentamicin cassette, Gm^r^	[36]
pTNS2	R6Kori vector carrying Tn7 transposase, Amp^r^	[37, 38]
pUC − 18 − miniTn7 LAC Apra	*E. coli*/*A. baumannii* shuttle expression vector with Ptac promoter and Tn7 insertion sequece, Apra^r^	[37, 38]
pGEM − T	Cloning vector, Amp^r^	Promega
pCR−BluntII−TOPO	Cloning vector, Km^r^, Zeo^r^	Invitrogen
pKOBEG	Vector containing the λ Red recombinase system, Cm^r^, temperature sensitive	[39]
pVRL1	Vector carrying a gentamicin cassette, Gm^r^	[40]
pUA1108	pGEX 4T–1 derivative plasmid lacking the GST fusion tag, containing only the Ptac IPTG–inducible promoter and the *lacIq* gene, Amp^r^	[41]

Amp^r^, Apra^r^, Km^r^, Gm^r^ and Zeo^r^ stand for resistance to ampicillin, apramycin, kanamycin, gentamicin and zeocin, respectively.

### Knockout mutant construction

For *A. baumannii*, the kanamycin − and zeocin−resistant plasmid pCR−BluntII−TOPO, which is incapable of replicating in *A. baumannii*, served as a suicide vector. An internal fragment (approximately 500 bp) of the target gene was PCR−amplified using specific oligonucleotides (Table S4) and genomic DNA from *A. baumannii* as a template. The resulting PCR product was cloned into the pCR−BluntII−TOPO vector and introduced into *E. coli*, generating the recombinant plasmid. Subsequently, the recombinant plasmid (0.1 μg) was introduced into the kanamycin − and zeocin−susceptible *A. baumannii* ATCC 17978 strain through electroporation, and mutants were selected on kanamycin−containing plates. Verification of target gene inactivation via plasmid insertion through single crossover recombination was confirmed by sequencing the amplified PCR products using the appropriate primer pairs (Table S4). For *E. cloacae*, ORF coding regions were replaced with a gentamicin antimicrobial cassette, amplified from pVRL1 vector, by using a one–step method for inactivation as previously described [39]. All constructions and mutants were confirmed by DNA sequencing.

### Mutant complementation

For the complementation of the mutant lacking the AdeB_Ab_ component, the gene *adeB*_*Ab*_ was amplified by PCR with oligonucleotides listed in Table S4 and cloned into the multicloning site of vector pBAV1Gm − T5 − gfp. To complement the mutants lacking the transcriptional regulator AdeR, and to avoid the multicopy effect, the *adeR*_*Ab*_ *and adeR*_*Ec*_ genes, along with their respective native RBSs (Ribosome Binding Sites), were introduced into the chromosome of the corresponding knockout mutant as follows. The genes were amplified by PCR using the primers listed in Table S4. The PCR products were cloned into the BamHI/KpnI sites of pUC18 − mini−Tn7 LAC Apra and transformed into competent *E. coli* λpir S17 cells by electroporation. A clone containing the plasmid with the insert in the correct orientation was identified by PCR. This plasmid was then cotransformed along with the pTNS2 plasmid into the corresponding mutant strain. Transformations were plated on LB agar containing apramycin. Transformants were screened by PCR for insertion of the wild−type gene into the *glmS* site. For the complementation experiments, IPTG was used at a concentration of 2 mM to induce expression.

### Analysis of antimicrobial susceptibility

The impact of the selected genes on antimicrobial resistance in the constructed mutants was analyzed as follows. Minimum inhibitory concentrations (MICs) for a representative number of antimicrobials from different families were determined by the Mueller Hinton broth microdilution method, according to CLSI (Clinical & Laboratory Standards Institute) guidelines. Briefly, standardized bacterial suspensions (5 ×  10^5^ CFU/mL) in a final volume of 0.1 mL were made in 96–well microtiter plates from the resuspension of colonies obtained from fresh culture plates. Once the inoculum of the corresponding culture was obtained with a turbidity comparable to 0.5 McFarland (approximately 10^8^ CFU/mL), a 1/100 dilution was performed to achieve a concentration of 10^6^ CFU/mL. By adding the medium containing the corresponding antimicrobial, a final inoculum concentration of 5 x 10^5^ CFU/mL was obtained. After incubation at 37 °C for 24 h, the turbidity was measured using the automatic reader Multiskan™ FC Microplate Photometer.

### Motility studies

Motility assays were performed as previously described [42]. Briefly, fresh LB agar motility plates (1% tryptone, 0.5% yeast extract, 0.5% NaCl, 0.5% glucose, and 0.5% Difco agar) were prepared the day of the assay. After sterilization, the medium was poured into 9–cm petri dishes with constant agitation to ensure the homogeneous spread of the agar. The inoculum was applied with a sterile toothpick. A single colony was picked and inoculated in the center of the plate. The inoculated plates were incubated at 37°C for 24 hours. All assays were carried out a minimum of three times in independent experiments.

### Virulence experiments

The *Galleria mellonella* (wax worm) model of virulence was used following the methodology previously described [43]. Briefly, 10 caterpillars (approximately 250 mg in weight) were used for each bacterial strain to be tested. Cells from bacterial cultures at the appropriate cell concentration were washed and resuspended in phosphate–buffered saline (PBS). The inoculum concentrations were confirmed by bacterial colony counts on agar plates. A 10–μL inoculum of the corresponding strain or 10 μL of PBS (as a negative control) was injected into the hemocoel of each worm through the last left proleg. The larvae were incubated at 37°C in the dark, and their survival was checked every 24 hours for a total of 96 hours. All *G. mellonella* killing experiments were performed at least twice. Survival curves were plotted using the Kaplan−Meier method and differences in survival were calculated using the log−rank test. In all cases, statistical significance was defined as a *P* value <  0.05.

### Protein purification

DNA fragments containing the target genes were amplified from purified chromosomal DNA of *A. baumannii* ATCC 17978 or *E. cloacae* ATCC 13047 by PCR with specific primers (Table S4). Following purification, the PCR products were enzymatically digested with the appropriate restriction enzymes, inserted into the corresponding restriction sites in the polylinker of the pUA1108 expression vector, and introduced into *E. coli* DH5α cells. The resulting recombinant plasmids were anticipated to express N − terminal His6 − tagged fusion proteins. Verification of correct in−frame fusions was conducted by sequencing plasmid DNA using COM_ pUA1108_F and COM_ pUA1108_R (Macrogen Sequencing Service). The validated recombinant plasmids were then employed to transform strain BL21 − CodonPlus(DE3) − RIL.

Each resulting BL21 − CodonPlus(DE3) − RIL strain was cultured overnight, diluted (1/20) in 10 mL of LB medium, and incubated at 37°C until the optical density at λ600 nm (OD_600_) reached 0.6. Induction of fusion protein expression was initiated by adding IPTG to a final concentration of 1 mM. After an additional 3 − hour incubation at 37°C, cells were harvested by centrifugation, resuspended in equilibration/wash buffer, and lysed by sonication. The resulting supernatant was mixed with BD TALON resin (Clontech), washed, and the proteins were recovered using 1 mL of elution buffer (50 mM sodium phosphate, 300 mM sodium chloride, 10 mM imidazole). The purified proteins were then dialyzed to remove imidazole, visualized by sodium dodecyl sulfate−polyacrylamide gel electrophoresis (SDS−PAGE) and quantified by the Bradford method.

### Electrophoretic mobility shift assays

DNA promoters were PCR−amplified from genomic DNA using appropriate oligonucleotides (Table S4), and the resulting PCR fragments were cloned into *E. coli* DH5α cells via the pGEM − T vector (Promega). The presence of the desired promoter was confirmed by sequencing the plasmid DNA using the M13FpUC and M13RpUC primers (Table S4). DNA probes were generated by PCR amplification, with one primer labeled with DIG at its 5′ end (Table S4). DNA−protein reaction mixtures (20 μL), consisting of 25 ng DIG−DNA−labeled probe and either 0 or 10 μg purified *A. baumannii* or *E. cloacae* regulatory protein, were incubated in EMSA buffer [20 mM Tris/HCl (pH 8), 50 mM KCl, 5% (v/v) glycerol, 1 μg bulk carrier salmon sperm DNA, 0.5 mM DTT, and 0.1 mg BSA mL^ − 1^] for 10 minutes at room temperature. DNA−protein complexes were visualized by electrophoresis on a 5% non−denaturing polyacrylamide gel (40 mM Tris/acetate) at 150 V for 1.5 hours and then transferred to a Biodine B nylon membrane (Pall Gelman Laboratory). DIG−DNA−labeled protein complexes were detected following the manufacturer’s protocol (Roche). When required, promoter fragments without DIG were used as unlabeled DNA competitors.

### RNA extraction and RT−PCR assays

An overnight culture of *E. cloacae* wild–type strain in LB medium was diluted 1:100 in fresh medium and then grown until the mid−exponential growth phase (OD_600_ =  0.4 − 0.6) was reached. The cells in 5 mL of each culture were pelleted by centrifugation at 13,000 ×  g, resuspended in Tris−EDTA (TE) buffer, and treated with lysozyme (50 mg/mL) for 10 min at 37°C. Total RNA was extracted using the RNeasy Mini kit (Qiagen). DNA contaminants were removed by digestion with TURBO DNA−free™ (Invitrogen). The absence of DNA in the RNA extractions was confirmed by PCR. RT−PCRs were performed using the appropriate oligonucleotides listed in Table S4 and the First−Strand cDNA Synthesis Kit (NZYtech) following the manufacturer’s instructions.

### Phylogenetic analysis

AdeB and AdeR homologs were compiled from all NCBI complete assemblies of *Acinetobacter* (n = 882), *Enterobacter* (n = 507) and from a randomly selected genome of each *Gammaproteobacteria* species (n = 1,372). Specifically, homolog prediction was performed through reciprocal BLASTP [44] using the *A. baumannii* ATCC 17978 AdeB (GenBank: ABO12177) and AdeR (Genbank: ABO12180) proteins sequences as the queries, a conservative e − value of < 1e–20 and query coverage of > 75%. All genomes and protein coding gene sequences were downloaded for %GC analysis.

For phylogenetic inference, we performed core genome alignments based on single nucleotide polymorphisms (SNPs) called by snippy (https://github.com/tseemann/snippy, accessed in October 2024). A Maximum Likelihood (ML) tree was constructed with iQtree [45] using 1000 bootstrap replicates and TVM + F + ASC + G as the substitution model, as deduced from ModelFinder [46]. Finally, ancestral state reconstruction was performed using RASP4 software, applying a Bayesian binary Markov Chain Monte Carlo (MCMC) approach, with the presence of the *adeB* and/or *adeR* genes set as the traits for analysis, and otherwise default parameters [47].

## Supporting information

S1 Table Prevalence of *adeB* and *adeR* in *Acinetobacter* spp.
(XLSX)

S2 Table Prevalence of *adeB* and *adeR* in *Enterobacter* spp.
(XLSX)

S3 Table Prevalence of *adeB* and *adeR* in other species of *Gammaproteobacteria.
* (XLSX)

S4 Table Oligonucleotides used in this work.
(DOCX)

S1 Fig Original uncropped and unadjusted images.
(PDF)
