# Supplementary material for: Reciprocal regulation between Acinetobacter baumannii and Enterobacter cloacae AdeR homologs: implications for antimicrobial resistance and pathogenesis
Source: PLoS One. 2025 Mar 10;20(3):e0315428. doi: 10.1371/journal.pone.0315428 (PMC11892822; doi:10.1371/journal.pone.0315428)
Supplement: S4 Table — (DOCX) [file pone.0315428.s004.docx]

**Table S4. Oligonucleotides used in this work.**

| **Name** | **Sequence 5′ 🡪 3′** | **Application** |
| --- | --- | --- |
| MUT_AdeRAbF | TCTG**GAATTC**TCTTGTGGTAGAAGATGACTAC | Mutant construction |
| MUT_AdeRAbR | TCTG**GAATTC**CTTTTTGGCGTATTTTATTTAACAC | Mutant construction |
| COM_ AdeRAbF | ATGTTTGATCATTCTTTTTCTTTTGATT | Mutant verification |
| COM_ AdeRAbR | TTAGGCGTCATCTTTTACAGC | Mutant verification |
| MUT_AdeBAbF | TCTG**GAATTC**CCGCCACAGGTGAATATTAG | Mutant consturction |
| MUT_ AdeBAbR | TCTG**GAATTC**TCGATAAACCGTAAGAAACAAGC | Mutant construction |
| COM_ AdeBAbF | ATGATGTCACAATTTTTTATTCGTC | Mutant verification |
| COM_ AdeBAbR | TTAAGATGAGATTTTTTTCTTAGAGGA | Mutant verification |
| MUT_AdeREcF | ATGTACAAAAATAATTTAATCCTAGTCGCCGAAGATGAAGATGAAATCGCCGATATACTGATGAGCTACCTGCAGCGGGCTTAGGTGGCGGTACTTGG | Mutant construction |
| MUT_AdeREcR | TCATTTCTTTTCTCCCAGCCGGTAGCCCATTCCTCTAATGCTTTCTGGCACACCATGAAGACCTGCAAGCTCCAGTTTTTGCGCGGAACCCCTATTTG | Mutant construction |
| COM_ AdeREcF | ATGTTTATAAAAAGTCGTCGCGC | Mutant verification |
| COM_ AdeREcR | GGAAGGTAATCGAGTACGAAG | Mutant verification |
| MUT_ AdeBEcF | ATGCCGCAATTTTTTATTGATCGTCCTGTTTTCGCCTGGGTCGTCGCACTTTTTATTGTTTTAACGGGCGTGTTGTCTATTTAGGTGGCGGTACTTGG | Mutant consturction |
| MUT_ AdeBEcR | TCACCTTTTTCTGGCGTGCTCGATGAAACGCGCGACGACAATAAAGAAAACCGGGACGAAGAAAATGGCGAGAAATGTACGCGCGGAACCCCTATTTG | Mutant construction |
| COM_ AdeBEcF | GCAACTCCTTGAGAAATTTTC | Mutant verification |
| COM_ AdeBEcF | GAATGAACGCGCGTAAGG | Mutant verification |
| pUC18_AdeRAbF | CAGT**GGATCC**CCTAGTGAGTTTTTGATGTTCG | Mutant complementation |
| pUC18_AdeRAbR | CAGT**GGTACC**TTAGGCGTCATCTTTTACAG | Mutant complementation |
| pBAV_AdeBAbF | CGTCGAATTCGCGGCCGCTTATGATGTCACAATTTTTTATTCG | Mutant complementation |
| pBAV_AdebAbR | ATAAATTTTTTATGATTTCCTTTAAGATGAGATTTTTTTCTTAGAG | Mutant complementation |
| pUC18_AdeREcF | CAGT**GGATCC**TTAATGAAAGCAATTGATAGACCGGG | Mutant complementation |
| pUC18_AdeREcR | CAGT**GGTACC**TCATTTCTTTTCTCCCAGCCGG | Mutant complementation |
| pUA1108_AdeBEcF | TTCACACAGGAAACAGTACAATGCCGCAATTTTTTATTGATC | Mutant complementation |
| pUA1108_AdeREcR | TCGACCCGGGGAATTCCGGGTCACCTTTTTCTGGCGTG | Mutant complementation |
| pUA1108_AdeREcF | TTCACACAGGAAACAGTACAATGTACAAAAATAATTTAATCCTAGTCG | *E. cloacae adeR* cloning for protein overexpression |
| pUA1108_AdeREchisR | TCGACCCGGGGAATTCCGGGTTAATGATGATGATGATGATGTCATTTCTTTTCTCCCAGCC | *E. cloacae adeR* cloning for protein overexpression |
| pUA1108_AdeRAbF | TTCACACAGGAAACAGTACAATGTTTGATCATTCTTTTTCTTTTG | *A. baumannii adeR* cloning for protein overexpression |
| pUA1108_AdeRAbhisR | TCGACCCGGGGAATTCCGGGTTAATGATGATGATGATGATGGGCGTCATCTTTTAC | A. baumannii adeR cloning for protein overexpression |
| pAdeRAb_F | ATACTGTCCAAACCTAGTGAG | *A. baumannii adeR* promoter amplification |
| pAdeRAb_R | AGAAAATCTGGCTATAGAAAGTGC | *A. baumannii adeR* promoter amplification |
| pAdeREc_F | ATGAAAGCAATTGATAGACCG | *E. cloacae adeR* promoter amplification |
| pAdeREc_R | TTATCTCCACAGGAACTGAAAG | *E. cloacae adeR* promoter amplification |
| RT1758F | AAAACAATAAAAAGTGCGACGAC | RT-PCR |
| RT1759F | ATTTTCGGCAATAACCAGAAAGA | RT-PCR |
| RT1759R | CAGCTCAGAGACATCGGTAAA | RT-PCR |
| RT1760R | TTGTTGGCTCACCCATTTATC | RT-PCR |
| M13FpUC* | GTTTTCCCAGTCACGAC | Sequencing primer for pCR-BluntII-TOPO and pGEM−T |
| M13RpUC* | CAGGAAACAGCTATGAC | Sequencing primer for pCR-BluntII-TOPO and pGEM−T |
| pBAV_F | CACTGTTCCTTGCATTCTA | Sequencing primer for pBAV1Gm-T5-gfp |
| pBAV_R | ATTGGGACAACTCCAGTGAA | Sequencing primer for pBAV1Gm-T5-gfp |
| pUC18_ComF | TAGATTTCACTTATCTGGTTGGC | Sequencing primer for pUC18−mini−Tn7 LAC |
| pUC18_ComR | CCTTTCGTCTTCACCTCGAG | Sequencing primer for pUC18−mini−Tn7 LAC |
| AB_glmSF | TATGGAAGAAGTTCAGGCTC | Verification pUC18−mini−Tn7 LAC Apra *glmS* insertion in *A. baumannii* |
| EB_glmSR | CATTGAAGAAGTTCGCGCCCG | Verification pUC18−mini−Tn7 LAC Apra *glmS* insertion in *E. cloacae* |
| Tn7R | CACAGCATAACTGGACTGATTTC | Verification pUC18−mini−Tn7 LAC Apra *glmS* insertion |
| COM_ pUA1108_F | CCGACATCATAACGGTTC | Sequencing primer for pUA1108 vector |
| COM_ pUA1108_R | AGACAAGCTGTGACCGTC | Sequencing primer for pUA1108 vector |

Sequences recognized by restriction enzymes are in bold and underlined (GAATTC: EcoRI, GGATCC: BamHI, and GGTACC: KpnI).

*When necessary, labelled with DIG at 5′ for EMSAs.
