# Supplementary material for: Reciprocal regulation between Acinetobacter baumannii and Enterobacter cloacae AdeR homologs: implications for antimicrobial resistance and pathogenesis
Source: PLoS One. 2025 Mar 10;20(3):e0315428. doi: 10.1371/journal.pone.0315428 (PMC11892822; doi:10.1371/journal.pone.0315428)
Supplement: S1 Fig — (PDF) [file pone.0315428.s005.pdf]

S1 raw images. ChemiDoc™ XRS+ System with Image Lab™ Software (Bio-Rad) was used to capture all the images shown in this document.

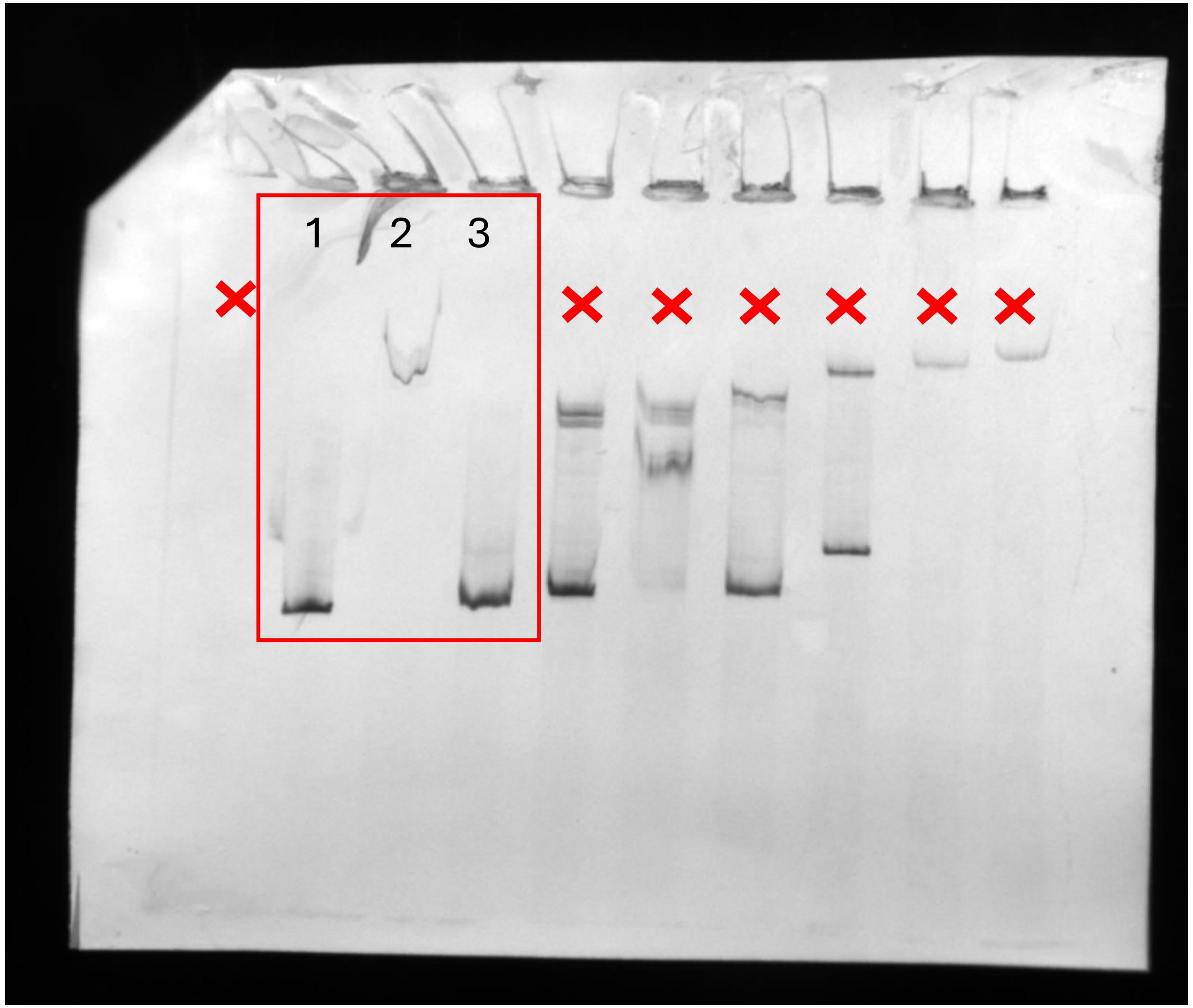

Blot used to generate Fig 3B.

NZYDNA Ladder V (NZYtech) was used as a molecular size marker (lanes 1 and 5)

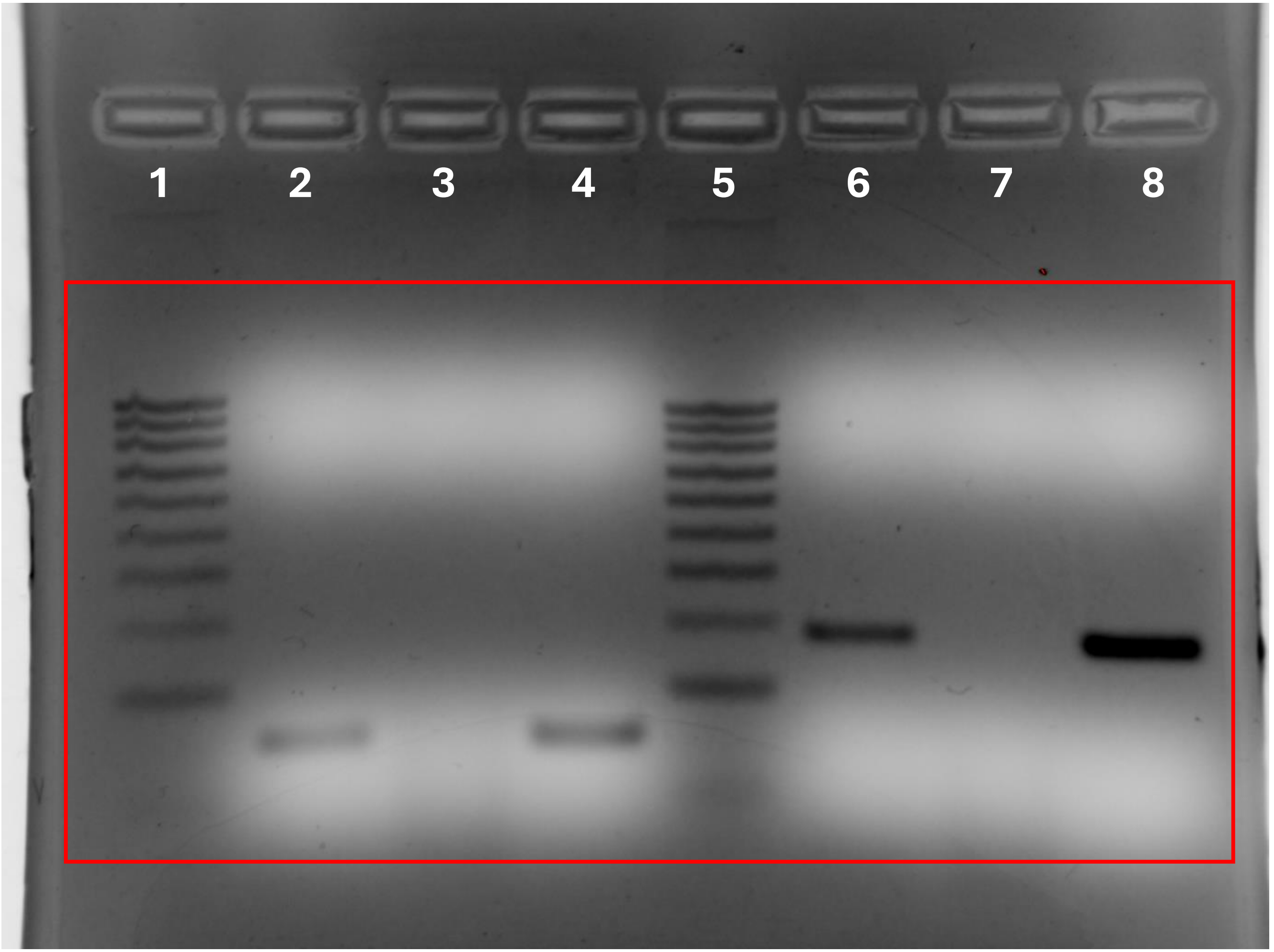

Gel used to generate Fig 4B.

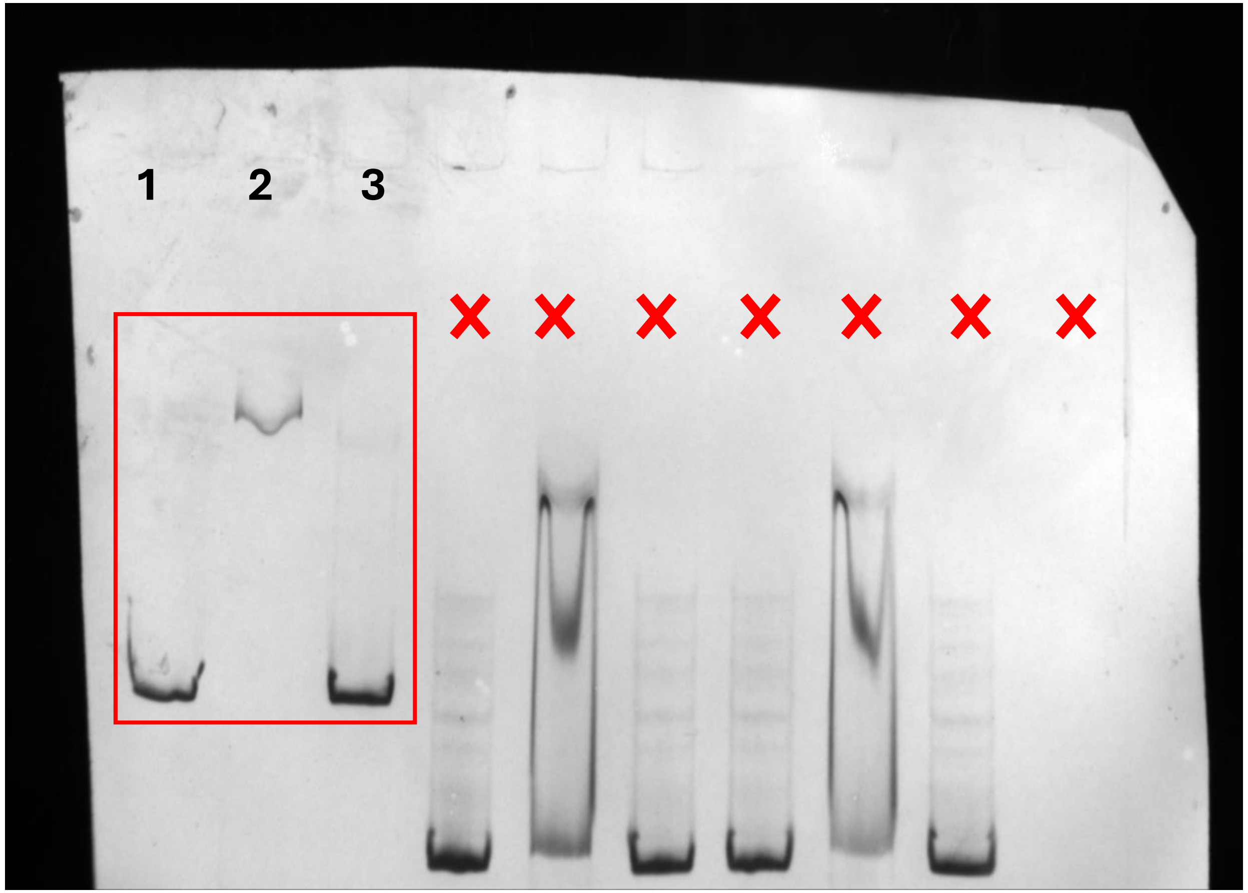

Blot used to generate Fig 4C.

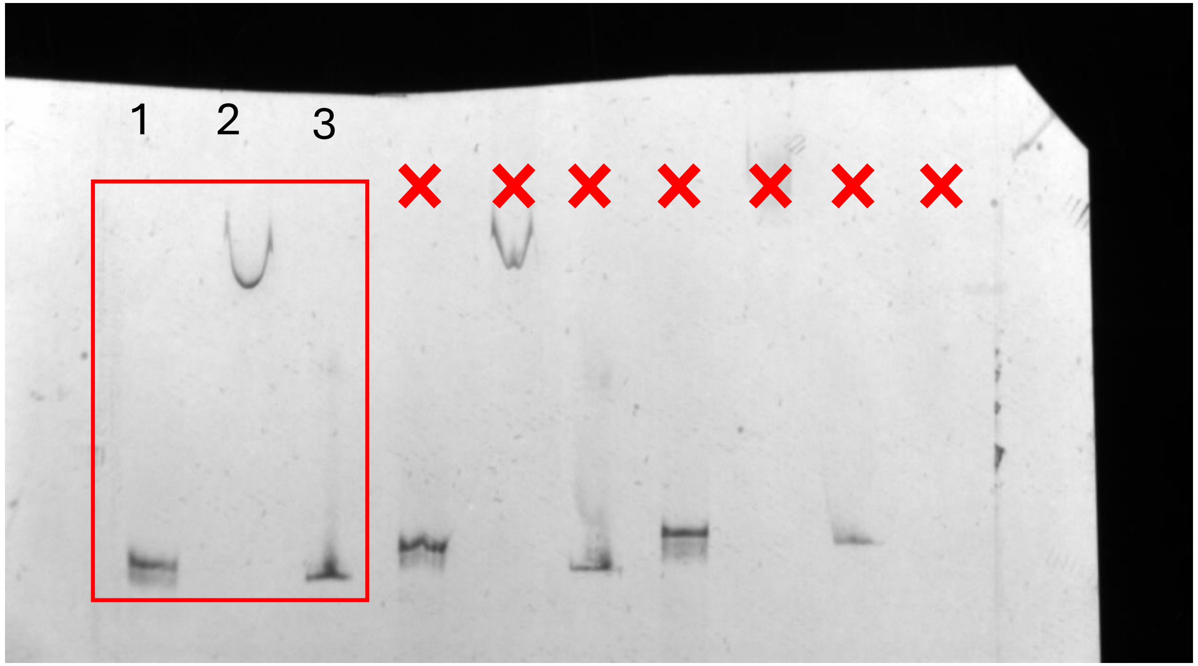

Blot used to generate Fig 5B.

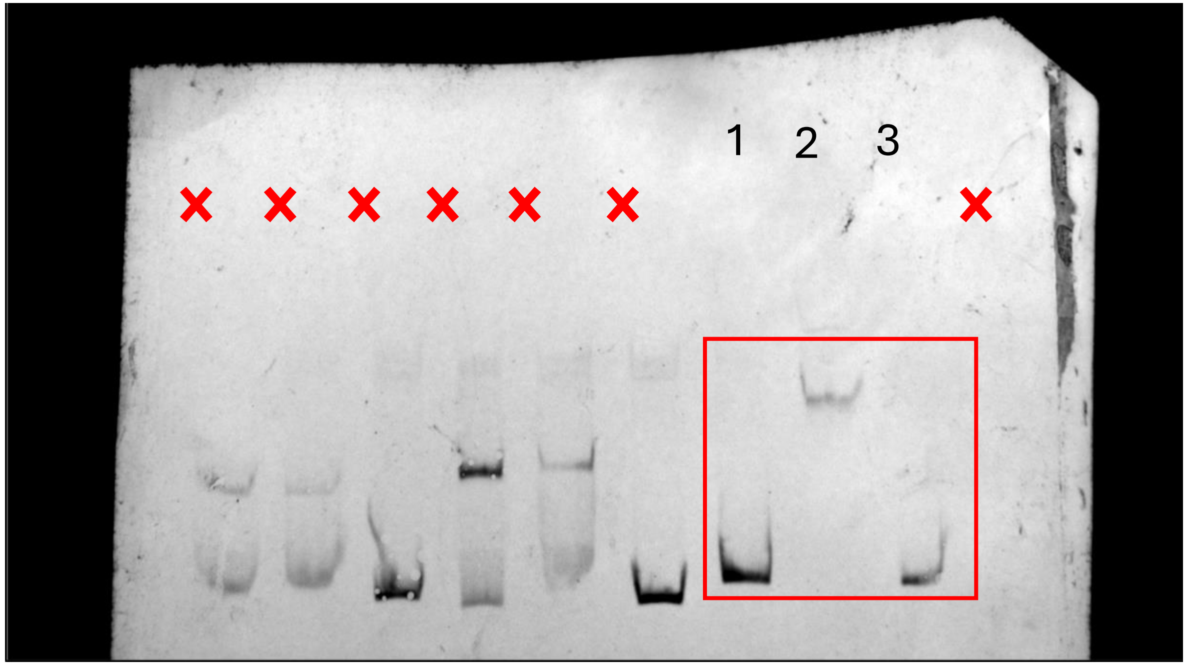

Blot used to generate Fig 5C.
